# Supplementary figures and images for: Lytic bacteriophages induce the secretion of antiviral and proinflammatory cytokines from human respiratory epithelial cells
Source: PLoS Biol. 2024 Apr 23;22(4):e3002566. doi: 10.1371/journal.pbio.3002566 (PMC11037538; doi:10.1371/journal.pbio.3002566)

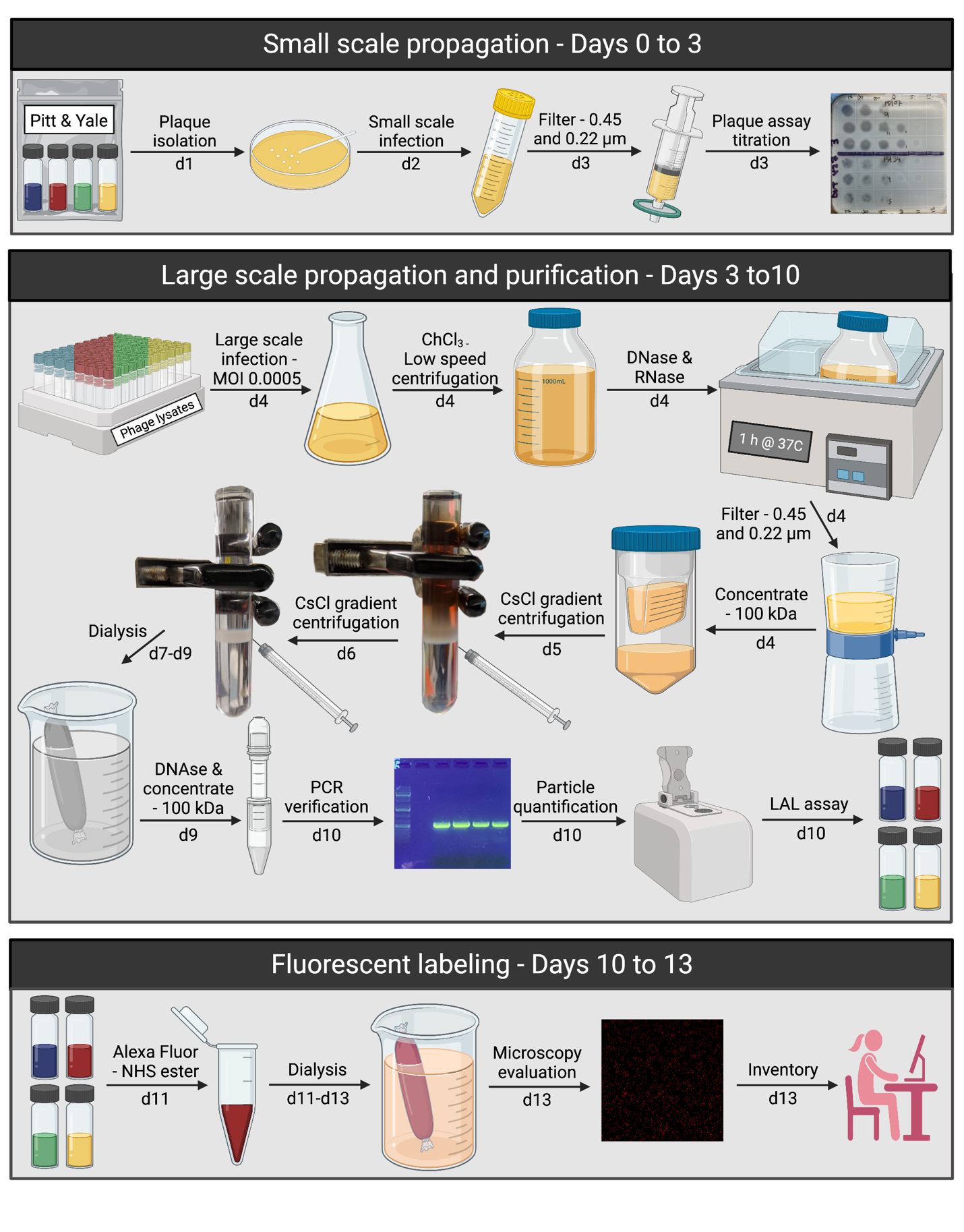

Supplement: S1 Fig — Bacteriophages OMKO1, LPS-5, PSA04, and PSA34 were shipped at 4°C and used in a small-scale propagation to generate phage lysates that were stored at 4°C. Before large-scale propagations, phages were re-tittered by plaque assay and used to infect large volume cultures of P. aeruginosa strains PAO1 (for phages OMKO1 and LPS-5), DVT423 (for PSA04), and DVT411 (for PSA34). Filtered and concentrated phage lysates were loaded onto CsCl gradients and phages were twice purified by gradient ultracentrifugation. Phage bands were collected, extensively dialyzed to remove CsCl, and phage identity was verified by PCR using phage-specific primers (S1 Table). Phage particles were quantified by absorbance at 269 and 320 nm. Endotoxin levels were quantified using the limulus amoebocyte lysate (LAL) test. Phages to be used in microscopy experiments were fluorescently labeled with Alexa Fluor dyes and fluorescence was confirmed before experiments by microscopy. Figure was created with BioRender.com. (TIF) [file pbio.3002566.s001.tif]

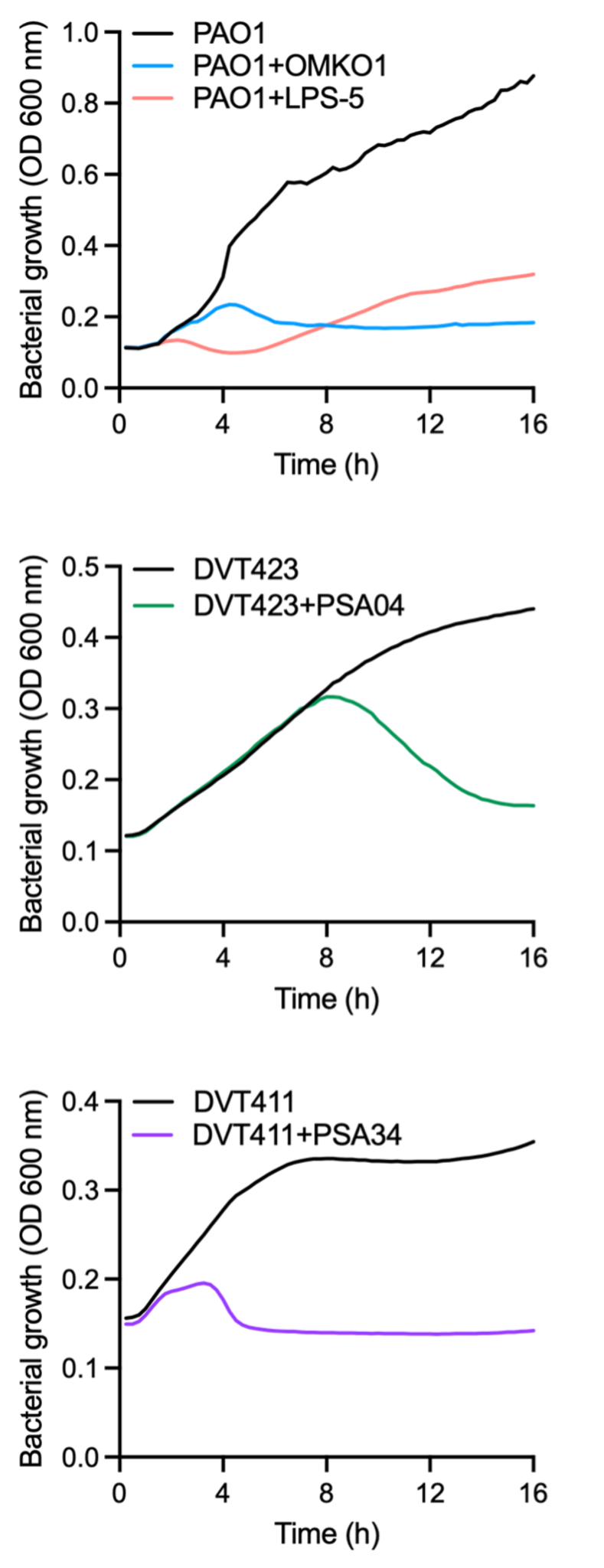

Supplement: S2 Fig — P. aeruginosa strains PAO1, DVT423, and DVT411 grown in LB broth were incubated with phages OMKO1 (PAO1), LPS-5 (PAO1), PSA04 (DVT423), and PSA34 (DVT411) at an MOI of 0.01 PFU/bacterium at 37°C. Bacterial growth over time was monitored by measuring absorbance at an optical density of 600 nm over 16 h. Results represent the mean from 3 independent experiments. The data underlying the panels in this figure can be found in S1 Data. (TIF) [file pbio.3002566.s002.tif]

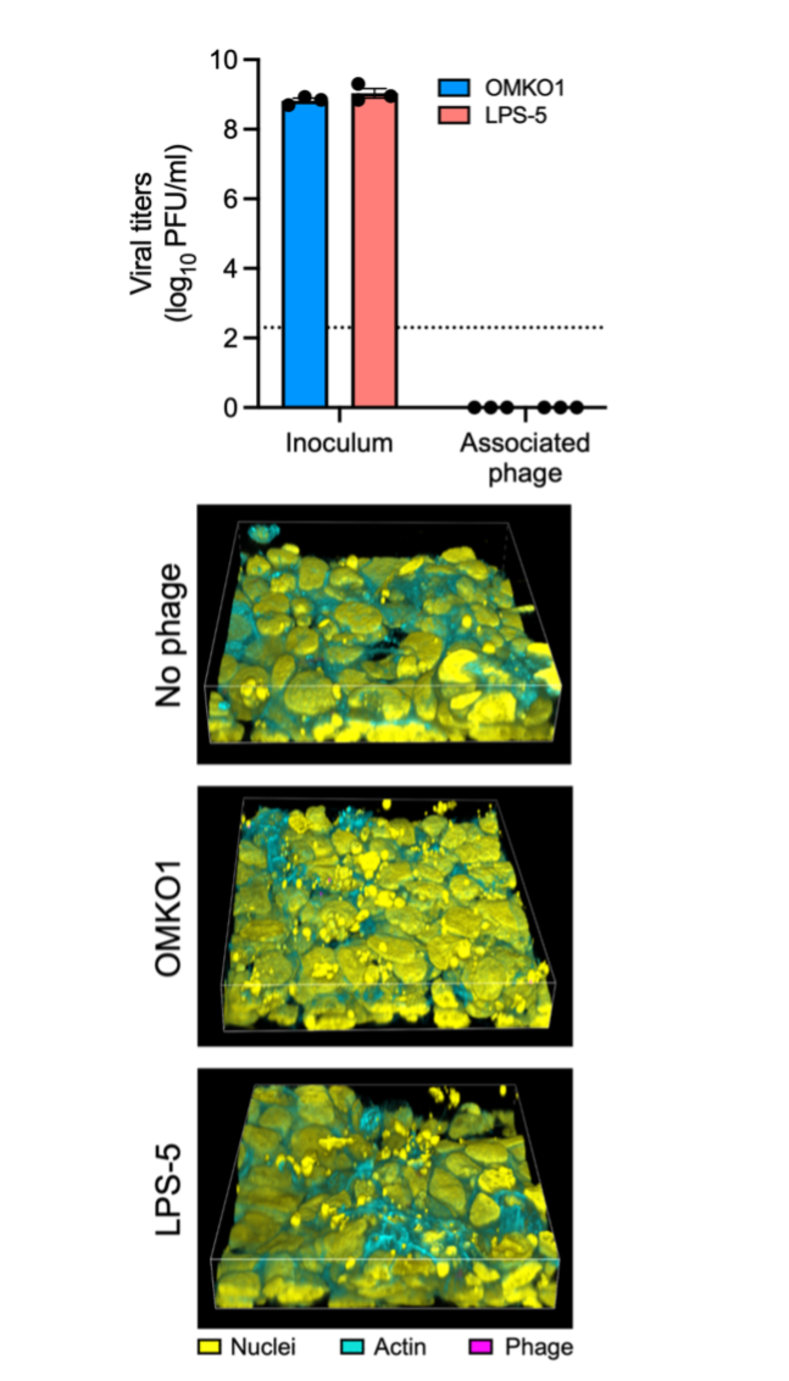

Supplement: S3 Fig — Unlabeled (top) and fluorescently labeled (bottom) OMKO1 and LPS-5 phages were incubated with CFBE41o- cells at an MOI of 1 × 10^9^ PFU/ml, following by immediate removal of the inoculum (0 h posttreatment). These phages were chosen for this experiment as they represent the phages with the highest and lowest internalized phage after a 1 h incubation (data from Fig 6). Inoculum and cell-associated phages were quantified by plaque assay (top). After inoculum removal, cells were fixed and processed for confocal microscopy (bottom). Images are shown as 3D reconstructions. LOD, limit of detection. Results represent the mean from 3 independent experiments. The data underlying the panels in this figure can be found in S1 Data. (TIF) [file pbio.3002566.s003.tif]

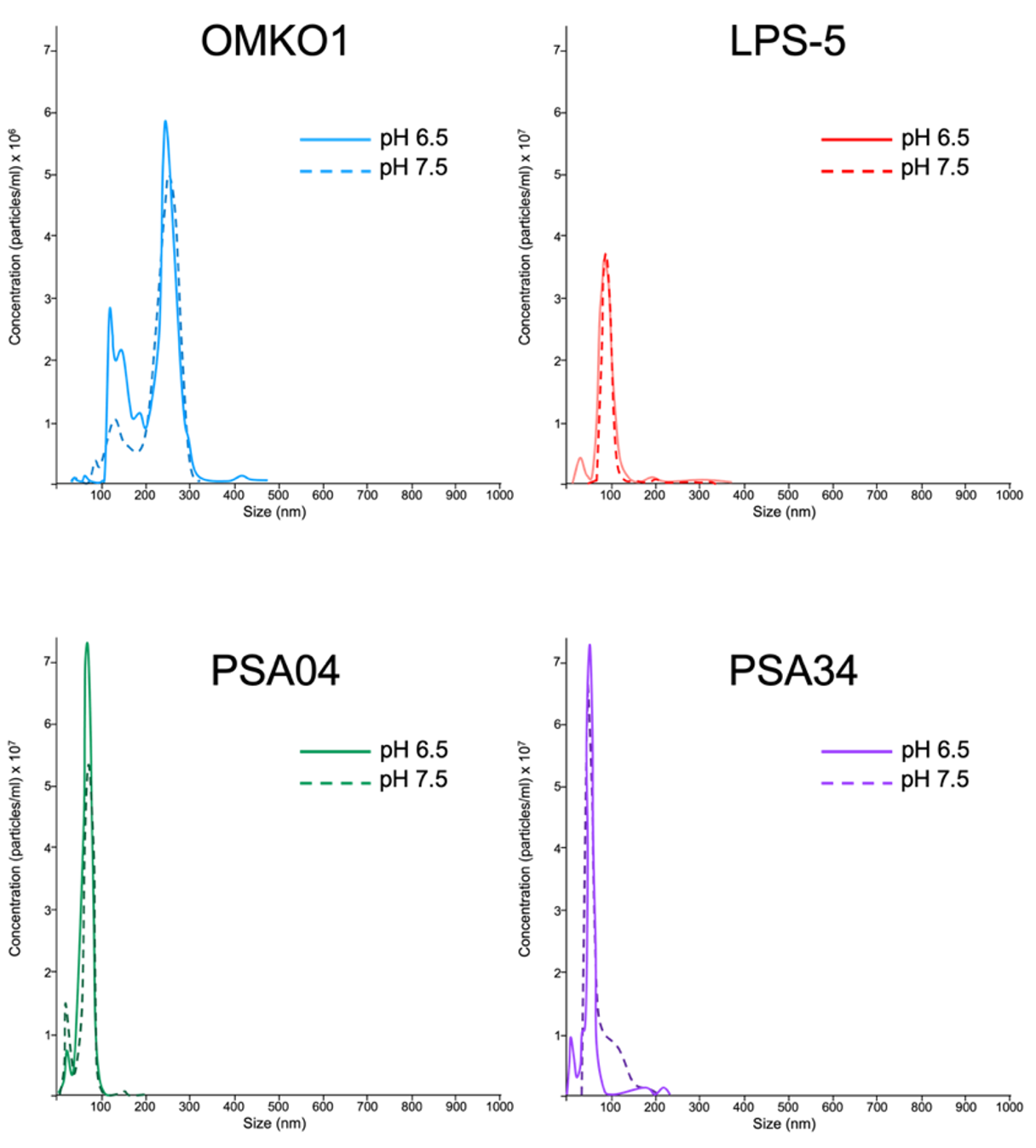

Supplement: S4 Fig — Phages OMKO1, LPS-5, PSA04, and PSA34 were incubated in minimum essential media at pH 6.5 or 7.5 at a concentration of 1 × 10^8^ PFU/ml at 37°C for 24 h. Particle dynamics was tracked using a nanoparticle analyzer. The figure shows a representative experiment from 2 independent replicates. (TIF) [file pbio.3002566.s004.tif]

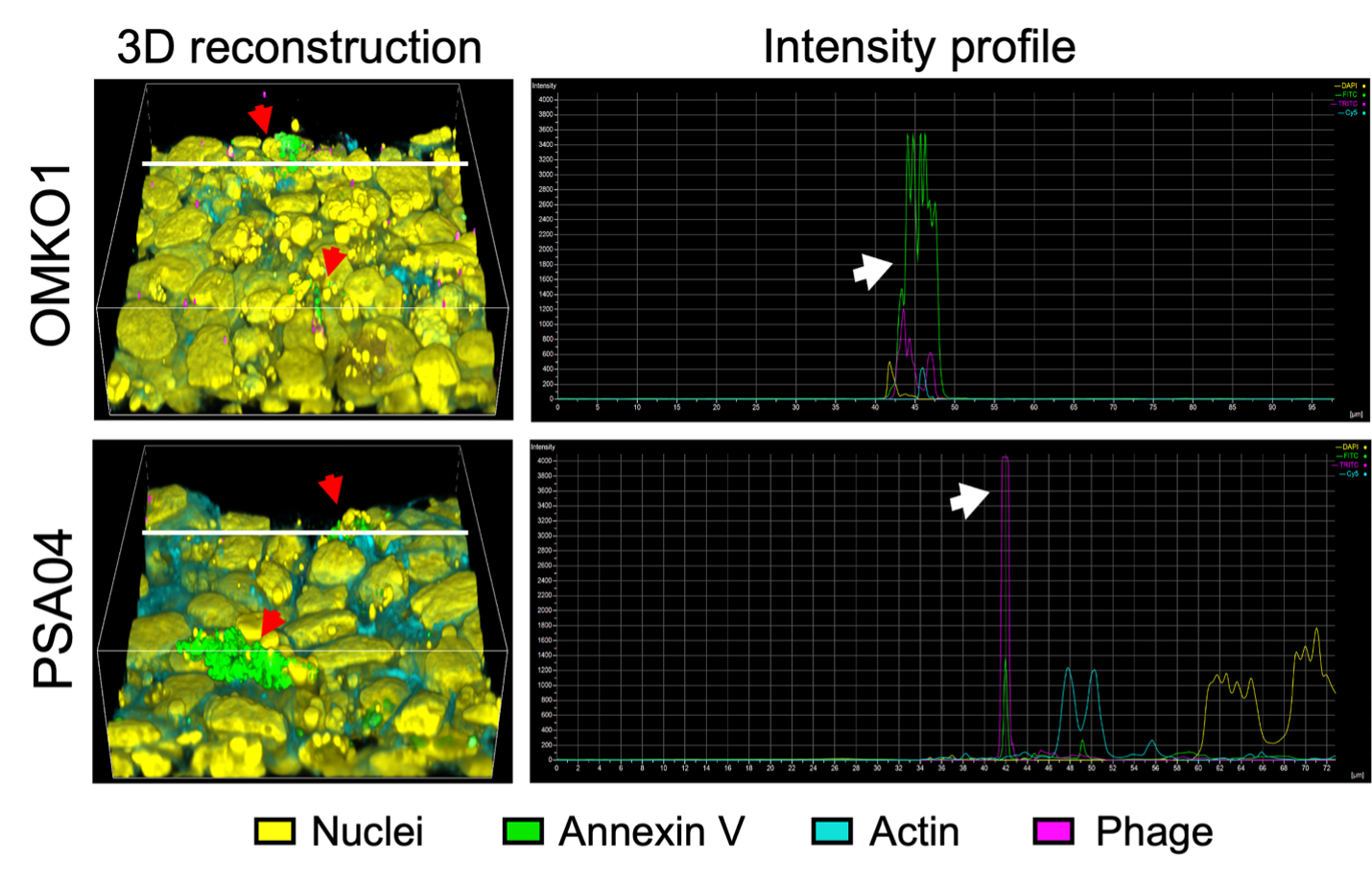

Supplement: S5 Fig — CFBE41o- cells grown in Transwell inserts were incubated with phages OMKO1 and PSA04 at an MOI of 1 × 10^9^ PFU/ml at 37°C for 1 h. These phages were chosen for this experiment as they represent phages with different interaction patterns with the airway epithelium. Cells were stained with annexin V to label apoptotic areas, fixed, and processed for confocal microscopy. Images show 3D reconstructions (left), red arrowheads indicate areas positive for annexin V staining. White line depicts the location in the XY dimension chosen for the intensity profiles (right), which portray fluorescence intensity over the X-axis. White arrows indicate areas of colocalization between annexin V and phage staining. The figure shows a representative experiment from 2 independent replicates. (TIF) [file pbio.3002566.s005.tif]

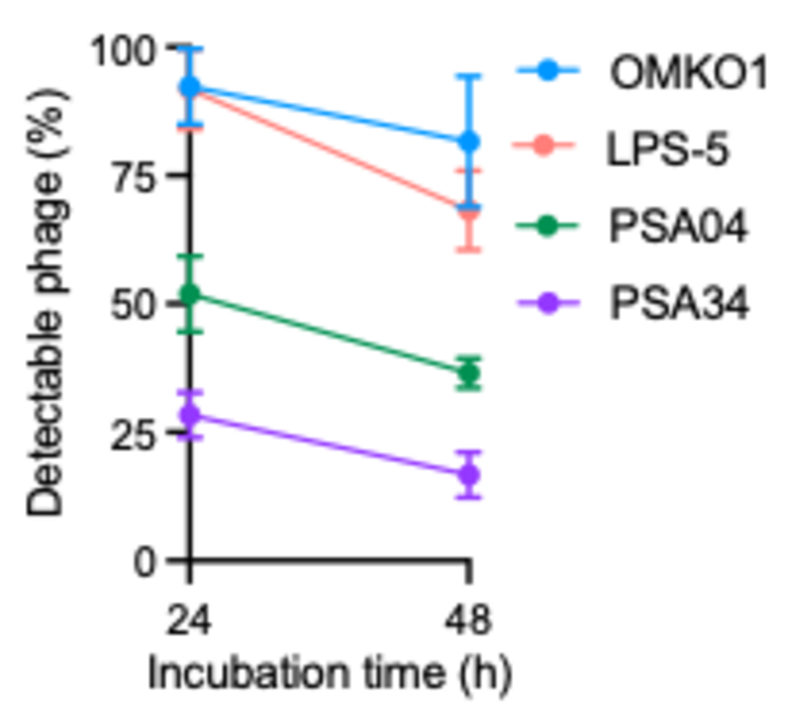

Supplement: S6 Fig — Phages OMKO1, LPS-5, PSA04, and PSA34 were incubated in tissue culture media at a concentration of 1 × 10^9^ PFU/ml at 37°C for 48 h. Phages were tittered at 0, 24, and 48 h after the start of the incubation. Results are shown as percentage titers at each time point compared to the titers at 0 h. Results show the mean of 3 independent biological replicates, with error bars indicating SEM. The data underlying the panels in this figure can be found in S1 Data. (TIF) [file pbio.3002566.s006.tif]

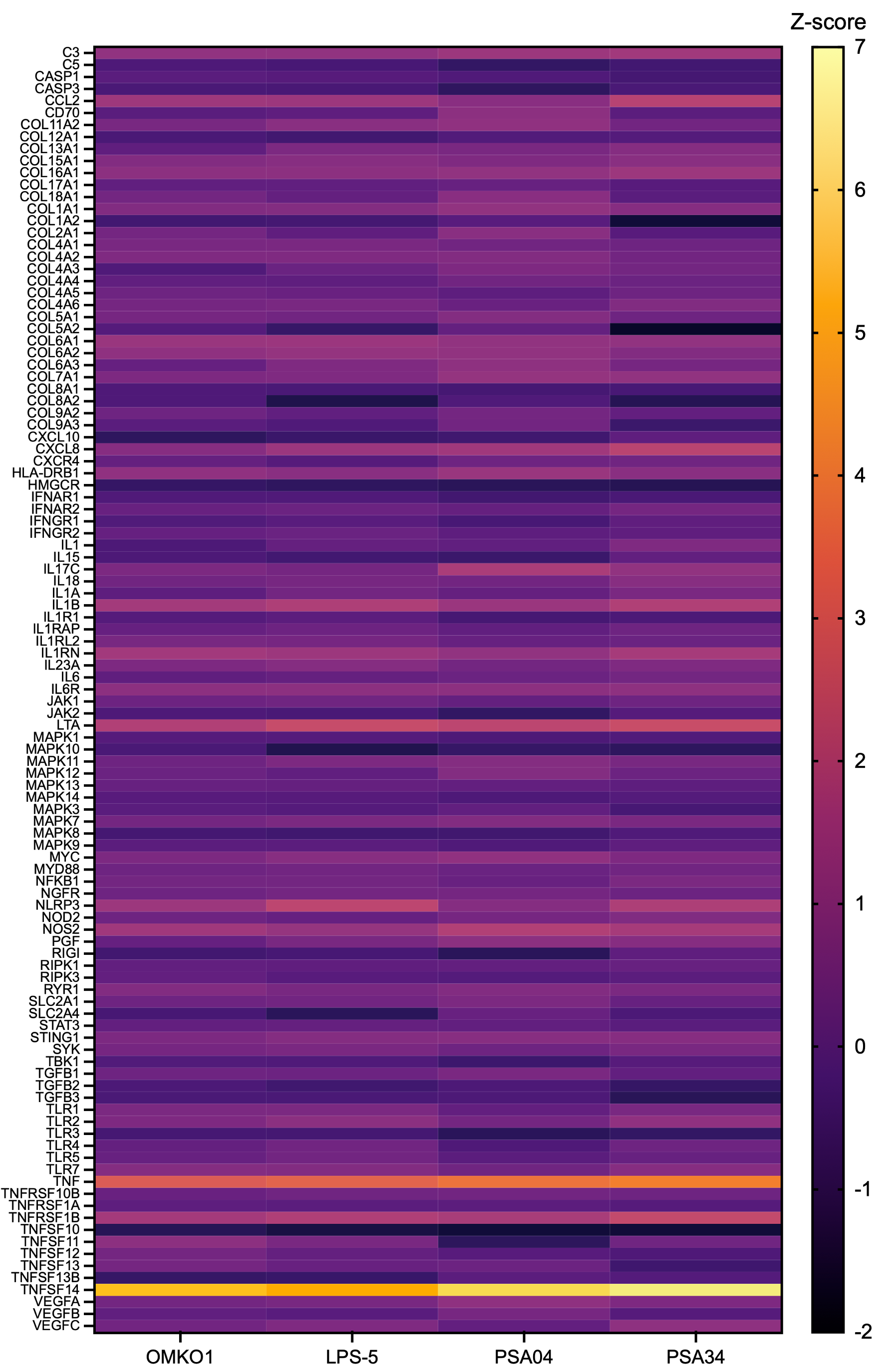

Supplement: S7 Fig — Individual Z-score values for genes in the “Pathogen Induced Cytokine Storm” pathway. Heatmap indicates Z-scores, as determined by the IPA z-score algorithm [92]. Z-scores were obtained from the differentially expressed genes shown in Fig 7, using RNA sequenced from at least 2 independent samples per treatment condition. The data underlying the panels in this figure can be found in S1 Data. (TIF) [file pbio.3002566.s007.tif]

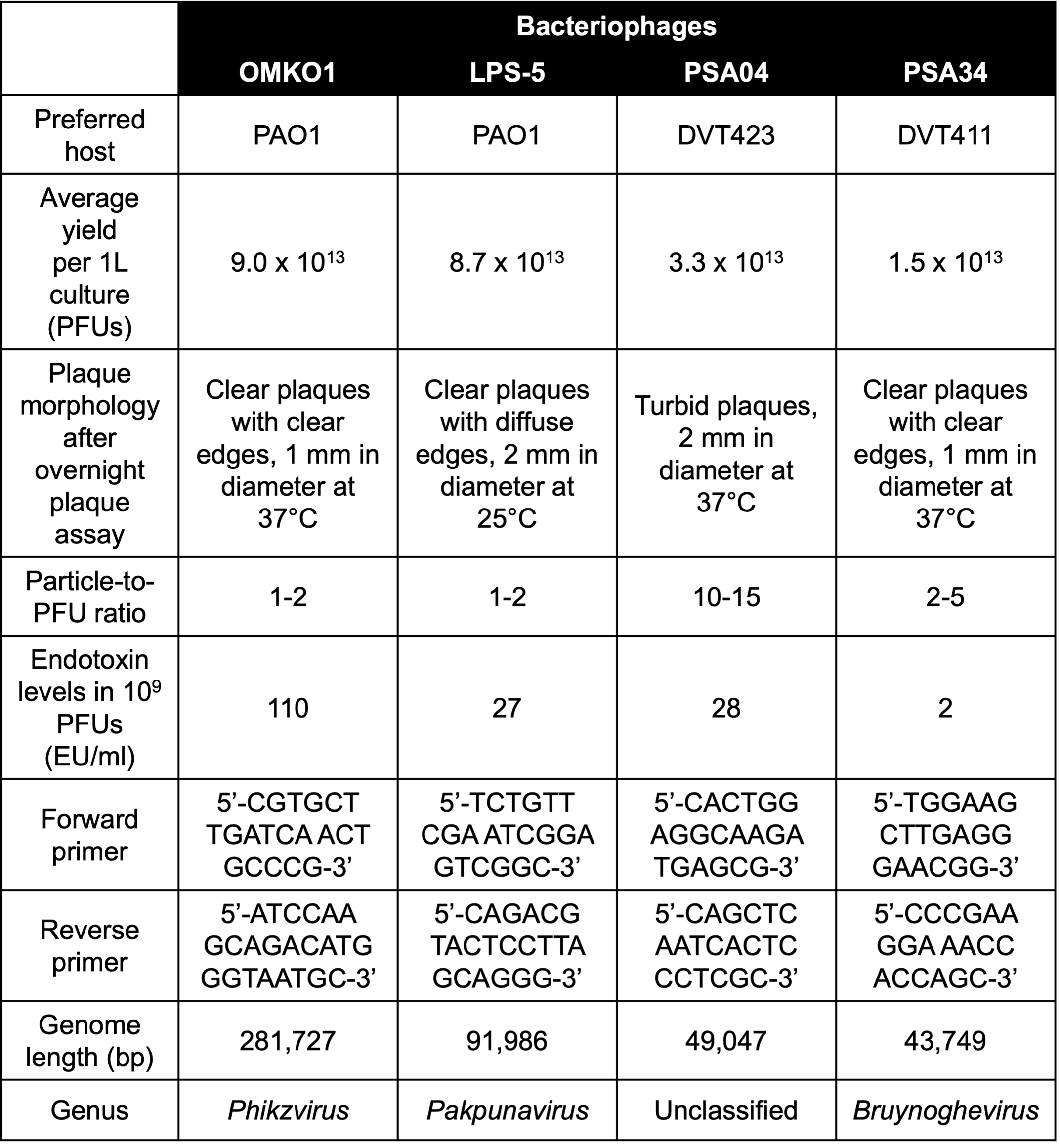

Supplement: S1 Table — (TIF) [file pbio.3002566.s008.tif]

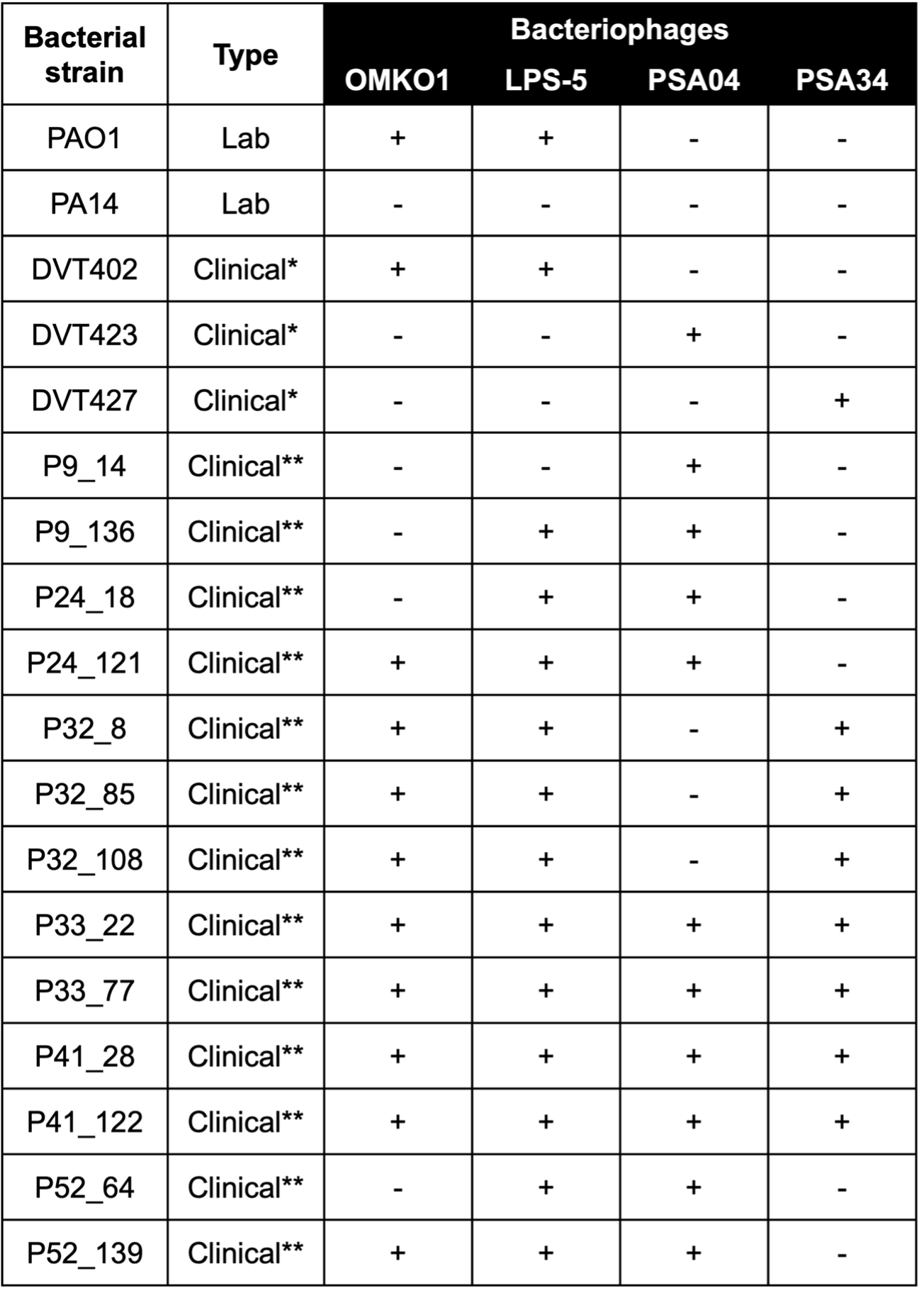

Supplement: S2 Table — Laboratory and clinical isolate strains of P. aeruginosa were tested for susceptibility towards phages OMKO1, LPS-5, PSA04, and PSA34 by plaque assay. “+” indicates susceptibility, “-”denotes resistance to the particular phage, *, clinical isolate strains obtained from CF sputum [24]; ** clinical isolate strains obtained from CF sinuses [74]. (TIF) [file pbio.3002566.s009.tif]
